# Human Milk Microbiome—A Review of Scientific Reports

**DOI:** 10.3390/nu16101420

**Published:** 2024-05-08

**Authors:** Agnieszka Dombrowska-Pali, Natalia Wiktorczyk-Kapischke, Agnieszka Chrustek, Dorota Olszewska-Słonina, Eugenia Gospodarek-Komkowska, Maciej W. Socha

**Affiliations:** 1 Department of Perinatology, Gynecology and Gynecologic Oncology, Faculty of Health Sciences, Collegium Medicum in Bydgoszcz, Nicolaus Copernicus University, Łukasiewicza 1, 85-821 Bydgoszcz, Poland; msocha@copernicus.gda.pl; 2 Department of Microbiology, Faculty of Pharmacy, Collegium Medicum in Bydgoszcz, Nicolaus Copernicus University, M. Curie-Skłodowskiej 9, 85-094 Bydgoszcz, Poland; n.wiktorczyk@cm.umk.pl (N.W.-K.); gospodareke@cm.umk.pl (E.G.-K.); 3 Department of Pathobiochemistry and Clinical Chemistry, Faculty of Pharmacy, Collegium Medicum in Bydgoszcz, Nicolaus Copernicus University, M. Curie-Skłodowskiej 9, 85-094 Bydgoszcz, Poland; a.chrustek@cm.umk.pl (A.C.); dorolsze@cm.umk.pl (D.O.-S.); 4 Department of Obstetrics and Gynecology, St. Adalberts’s Hospital in Gdańsk, Copernicus Healthcare Entity LLC, Jana Pawła II 50, 80-462 Gdańsk, Poland

**Keywords:** breastfeeding, human milk, microbiome, infant

## Abstract

One of the most important bioactive components of breast milk are free breast milk oligosaccharides, which are a source of energy for commensal intestinal microorganisms, stimulating the growth of *Bifidobacterium*, *Lactobacillus*, and *Bacteroides* in a child’s digestive tract. There is some evidence that maternal, perinatal, and environmental-cultural factors influence the modulation of the breast milk microbiome. This review summarizes research that has examined the composition of the breast milk microbiome and the factors that may influence it. The manuscript highlights the potential importance of the breast milk microbiome for the future development and health of children. The origin of bacteria in breast milk is thought to include the mother’s digestive tract (entero-mammary tract), bacterial exposure to the breast during breastfeeding, and the retrograde flow of breast milk from the infant’s mouth to the woman’s milk ducts. Unfortunately, despite increasingly more precise methods for assessing microorganisms in human milk, the topic of the human milk microbiome is still quite limited and requires scientific research that takes into account various conditions.

## 1. Introduction

In recent years, the World Health Organization (WHO) has recommended exclusive breastfeeding until the child is 6 months old and continuing with complementary feeding until the age of 2 or longer if, due to the health-promoting properties of human milk, the mother and child need it [1].

Natural feeding ensures the child’s optimal health and promotes proper development. Infants fed with human milk are less likely to suffer from infectious diseases, necrotizing enterocolitis, and sudden infant death syndrome (SIDS), die less often, and are additionally at a lower risk of developing the following in the future: overweight, obesity, type I and II diabetes, asthma, lymphoma, Hodgkin’s disease, lymphocytic leukemia, and myeloid hypercholesterolemia [1,2,3,4]. Breastfeeding also protects the mother. The immediate benefits of breastfeeding include a shorter period of bleeding after childbirth, faster uterine involution, lactation infertility, faster weight loss, and a lower level of depression [1,5]. However, it also has long-term beneficial effects, including a lower risk of breast cancer, ovarian cancer, osteoporosis, as well as a lower incidence of hip fractures, hypertension, diabetes, and hyperlipidemia [1,6].

Human milk is the mother’s most valuable gift to her child due to the health-promoting properties resulting from its composition. It is a colloidal solution of carbohydrates, fats, proteins, vitamins, growth factors, microelements, and other biologically active chemical compounds, the composition of which changes depending on the phases of lactation, the health of women, their physiology, race, or the geographical environment in which they live. Diet minimally affects the content of human milk components [7,8,9]. One of the most important bioactive components of human milk are free human milk oligosaccharides (HMOs), a type of prebiotic. HMOs are an energy source for commensal intestinal microbes. They stimulate the growth of *Bifidobacterium*, *Lactobacillus*, and *Bacteroides* in the infant’s digestive tract and are also believed to prevent neonatal diarrhea and respiratory infections [10,11]. The concentration of HMOs ranges from 20 to 25 g/L in colostrum and from 5 to 20 g/L in mature milk, which makes them the third-largest solid component in human milk [10,12].

The nutritional properties of human milk have been known for hundreds of years. Currently, human milk is treated as a medicine and has many clinical applications. This is related to its numerous health properties, i.e., anti-inflammatory, immunomodulatory, antioxidant, anti-infective, as well as its impact on the development of the digestive system [13,14,15,16,17,18,19,20,21].

**Figure 1:**
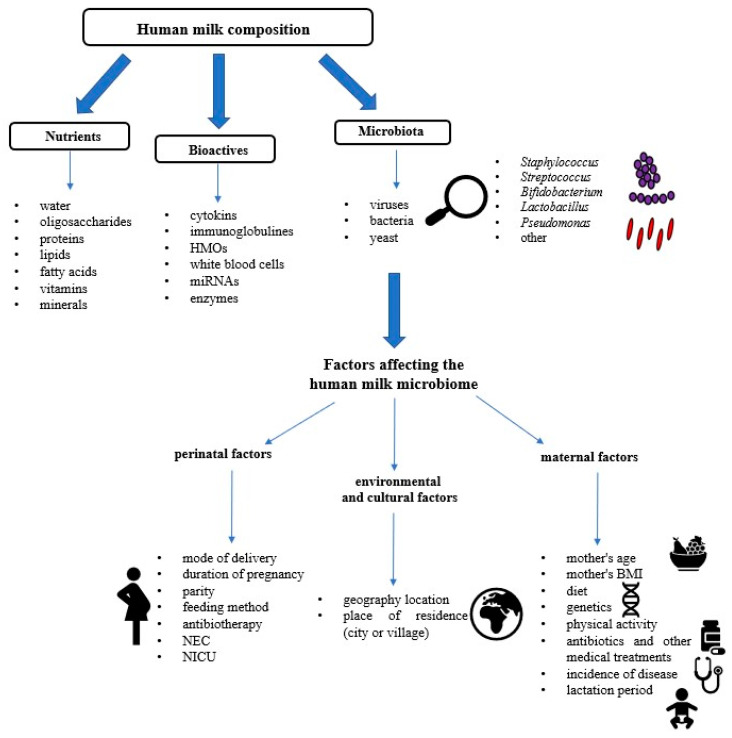
Basic composition of human milk and factors affecting the microbiome present in milk (according to [25,26]). (HMOs—human milk oligosaccharides; HAMLET—human a-lactalbumin made lethal to tumor cells; BMI—body mass index; NICU—neonatal intensive care unit; NEC—necrotizing enterocolitis).

In addition to a number of substances and nutrients, human milk contains a wide range of microorganisms [22] (Figure 1). The dominant bacteria in breast milk belong to the genera *Streptococcus*, *Staphylococcus*, *Micrococcus*, *Lactococcus*, *Lactobacillus*, and *Bifodobacterium* [17]. Consumption of breast milk has a positive effect on the colonization of the child’s intestinal microbiota, with a predominance of the genera *Bifidobacterium* and *Lactobacillus*. In turn, in children fed with formula milk, it was shown that there are fewer bifidobacteria and lactobacilli in the microbiota, despite the fact that the child had contact with these bacteria during vaginal delivery. Please note that microorganisms present in milk may play an important role in the development of the gastrointestinal microbiome of infants [23,24]. The transfer of many lactic acid bacteria from a breastfeeding mother is a natural mechanism that improves the development of the infant’s intestinal microbiota, which is associated with increased immunity [25]. The composition of the breast milk microbiome has a huge impact on the health of the baby, which is presented later in the manuscript. Therefore, considering the benefits of breastfeeding and the possible difficulties that breastfeeding women may experience, every effort should be made to support women during lactation. The role of midwives is crucial at this time because, as specialists in breastfeeding promotion, they have a great influence on mothers’ decisions about how to feed their children. Midwives are responsible not only for providing specialist help but also for increasing the sense of competence in their abilities when breastfeeding.

**Figure 2:**
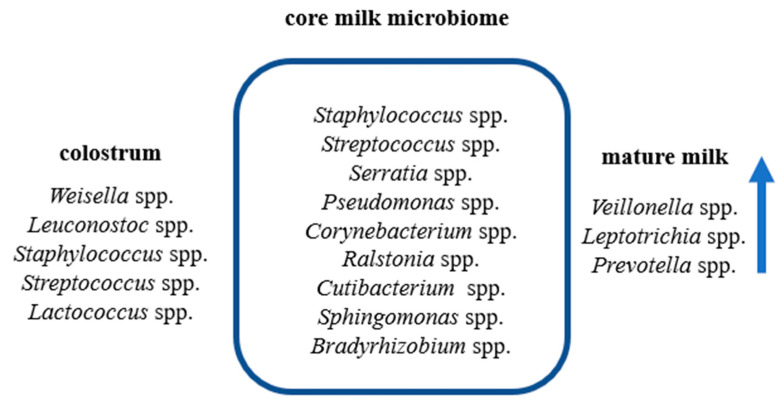
Core human milk microbiome and the dominant species in colostrum and mature milk.

Recent research by Boix-Amorós et al. [27], using molecular techniques, estimated that an infant may consume approximately 10^7^ to 10^8^ bacterial cells per day through mother’s milk (~800 mL). However, can we define the basic composition of human milk? Previous research has shown the presence of viruses, bacteria, and fungi in human milk [22]. The dominant types of bacteria have been distinguished, but the composition of milk is variable and depends on many factors (Figure 1 and Figure 2). So far, it has been shown that the “core” microbiome of human milk consists of the following genera: *Streptococcus*, *Staphylococcus*, *Serratia*, *Pseudomonas*, *Corynebacterium*, *Ralstonia*, *Cutibacterium*, *Sphingomonas*, and *Bradyrhizobium*, and their abundance varies depending on the sample (Figure 2) [28,29,30,31]. Metagenomic analysis of human milk by sequencing performed by Ward et al. [32] showed that human milk contains over 360 prokaryotic genera, including *Proteobacteria* (65%) and *Firmicutes* (34%) as the dominant types and *Pseudomonas* spp. (61.1%), *Staphylococcus* spp. (33.4%), and *Streptococcus* spp. (0.5%) as the dominant bacterial species. It should be emphasized that a normal microbiome also includes sequences related to fungi, protozoa, and viruses [33]. According to McGuire MK and McGuire MA [34], human milk is probably mother nature’s prototypical probiotic food—providing a cocktail of microorganisms, the amount of which depends on various factors and is necessary for the newborn during the critical period of growth and development.

## 2. Origin of Microbiota in Human Milk

**Figure 3:**
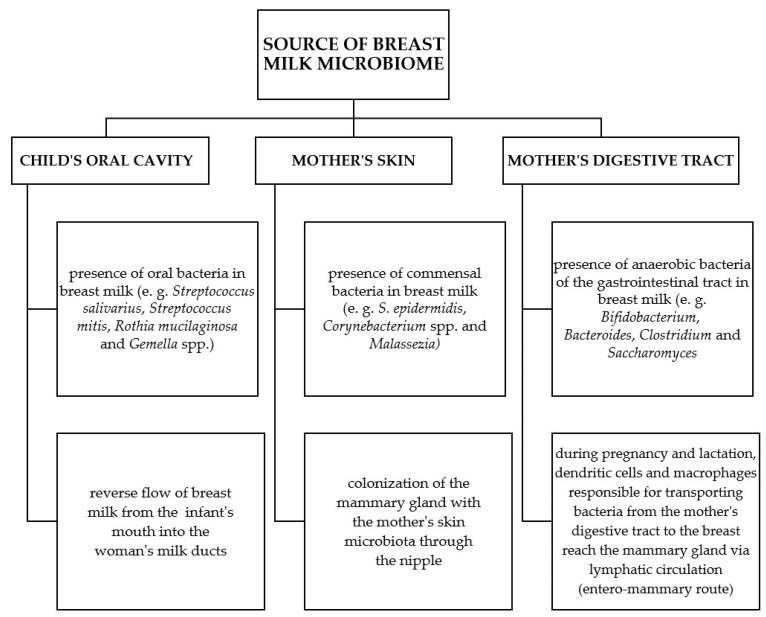
Overview of hypothetical sources of the human milk microbiome. Data is from references [22,27,35,36,37,38].

Colonization of human milk with microorganisms is a dynamic, complex, and not fully understood process. Until recently, it was believed that every bacterial cell present in human milk was the result of contamination of the mother’s skin or the baby’s oral cavity. Only the detection of live bacterial cells or DNA of anaerobic species in human milk, which cannot survive in aerobic conditions and are usually found in the intestinal environment, initiated a discussion among researchers on the hypothetical sources of the origin of bacteria in human milk [35], as shown in Figure 3. Research is still on going to determine whether the mammary gland is home to the so-called “mucosal surface model” or the “continuous flow model”.

### 2.1. Child’s Mouth Cavity

By analyzing the retrograde flow of human milk from the baby’s mouth into the milk ducts, it is likely that microorganisms from the baby’s mouth can seed the human milk bacterial microbiome. This hypothesis is supported by the presence of bacteria in human milk that are characteristic of the oral environment: *Streptococcus salivarius*, *Streptococcus mitis*, *Rothia mucilaginosa*, and *Gemella* spp. [22]. The importance of direct breastfeeding and the reverse flow of human milk from the baby’s mouth to the milk ducts was presented in the study of Biaga et al. The study included prematurely born children (gestational age 32–34 weeks) who initially received human milk from a bottle. Only after the child’s general condition and proper sucking function were stabilized did the children begin to be fed directly from the breast. It was observed that the human milk microbiome after the transition from feeding with expressed human milk using a bottle to direct breastfeeding became more diverse and dominated by typical oral microorganisms, i.e., *Streptococcus* and *Rothia* spp., thus confirming that contact with the baby’s oral cavity shapes the human milk microbiome [36]. However, research by Ruiz et al. [39] presents the opposite situation, in which oral bacteria found in human milk colonize the child’s oral cavity. The authors of this study found that there is a very large share of taxa in common between colostrum obtained from pregnant women before delivery and samples collected from the oral cavity of a newborn. Interestingly, in 8 of 10 pairs (mother–child), both isolates were > 99.9% identical at the nucleotide level. The results of these studies suggest that at least some of the bacteria typical of the oral cavity (the largest percentage of *Streptococcus* spp. and *Staphylococcus* spp.) enter the child’s oral cavity during breastfeeding. These data strongly suggest that some bacteria colonize the infant’s oral microbiome from human milk [39]. It is very likely that human milk bacteria and baby’s oral bacteria exchange characteristics, thus creating both environments during breastfeeding. According to the literature, the oral microbiome of breastfed children differs significantly from the oral microbiome of children fed with formula. These studies highlight that the choice of how to feed a child influences the development of the oral microbiome, which may ultimately impact both short- and long-term health effects [40].

### 2.2. Mother’s Skin

Human milk contains many bacteria of the *Staphylococcus* genus, including commensal bacteria typical of human skin: *S. epidermidis*, *S. hominis*, *S. haemolyticus*, and *S. lugdunensis*. Jiménez et al. [33], conducting research on 23 women and their children (16 breastfed and 7 fed with formula), showed that *S. epidermidis* dominated both in human milk and in the feces of breastfed infants. In turn, in infants fed with formula milk, *S. epidermidis* was less prevalent, which was a feature that differentiated both study groups [41]. Human skin commensal bacteria such as *Cutibacterium acnes* and species of the *Corynebacterium* genus are also often identified in human milk. Moreover, the human skin commensal *Malassezia* spp. is the main genus of fungi present not only in human milk, but also in and around the sebaceous glands [42]. Therefore, it can be assumed that the bacterial microbiota of the skin colonize the mammary gland by passing through the nipple. Pannaraj et al., in their research on a group of 107 healthy mother–child pairs, proved that 27.7% of gastrointestinal bacteria came from human milk, and another 10.3% from the skin of the areola [43].

It is certainly necessary to continue research in this area to assess the contribution of the mother’s skin bacterial microbiota to the microbiome of the child’s gastrointestinal tract and to the microbiome of human milk.

### 2.3. Maternal Digestive Tract

The literature on the subject indicates that the microbiome of human milk has common features with the microbiome of the gastrointestinal tract of a breastfeeding mother. The genus *Saccharomyces*, which includes some of the most abundant fungi identified in the gastrointestinal tract, is also one of the main types of fungi present in human milk [44]. Human milk also contains the following types of bacteria: *Bifidobacterium*, *Veillonella*, *Bacteroides*, *Parabacteroides*, *Clostridium*, *Collinsella*, *Faecalibacterium*, *Coprococcus*, and *Blautia* [33,45,46,47,48,49,50]. These bacteria are anaerobes that would not survive in the aerobic conditions of the child’s mouth and on the mother’s skin. Hence, the hypothetical “entero-mammary route”, which would explain the presence of the previously mentioned bacteria in human milk. Dendritic cells take up live bacteria by disrupting the intestinal epithelium. Then, for several days in the mesenteric lymph nodes, these cells sequester live bacteria, which are transferred through the lymphatic system to individual parts of the body, including the mammary gland [22]. Perez et al. [51], in studies on a group of mice, showed that bacteria could be identified 60% more often in the mesenteric lymph nodes of pregnant mice compared to mice that were not pregnant. The obtained results suggest that the ability of bacteria to translocate increases during lactation [51]. During this period, the number of lymphatic vessels in the mammary gland tissues increases. This is confirmed by research conducted on a group of mice that were orally administered two genetically modified strains of lactic acid bacteria (*Lactococcus lactis* MG1614 and *Lactobacillus salivarius* PS2). These bacteria were detected in milk and mammary tissue in a group of pregnant mice but not in a control group [52]. Subsequent studies indicate that some species of bacteria typical of the gastrointestinal tract (*Escherichia coli*, *Faecalibacterium*, and *Eubacterium* spp.) occasionally enter the mammary gland. Hence, the conclusion that only some bacteria have the ability to get from the digestive tract to the mammary gland [51]. It is also important that the microbiome of human milk can be modified, e.g., by diet or pre/probiotics used by a breastfeeding woman. Jiménez et al. [33] used a daily oral dose of *Lactobacillus salivarius CECT5713* and *Lactobacillus gasseri CECT5714* or a placebo in a group of breastfeeding women with diagnosed mastitis. At the beginning of the study, the average number of staphylococci in human milk in both groups was comparable (probiotic group 4.74 and control group 4.81 log_10_ colony forming unit (CFU)/mL). However, on the 30th day of the study, the average number of *Staphylococcus* spp. in the probiotic group (2.96 log_10_ CFU/mL) was significantly lower than in the control group (4.79 log_10_ CFU/mL), and *L. salivarius CECT5713* and *L. gasseri CECT5714* could be isolated from human milk samples in 6 out of 10 cases. Moreover, on day 14 of the study, no symptoms of mastitis were observed in the group of women using probiotics, and in women from the control group, symptoms of mastitis persisted throughout the study period [53]. In turn, when it comes to the impact of a breastfeeding woman’s diet on the microbiome of human milk, it has been proven that the gastrointestinal microbiota of breastfed children depends on the mother’s vegetarian diet [54].

## 3. Microorganisms in Human Milk

The microbiome of human milk is associated with a large bacterial diversity; the normal microbiome also includes sequences associated with viruses, fungi, and protozoa [33]. To date, data on the composition of human milk are related to the use of classical microbiological methods. However, the development of molecular biology techniques has made it possible to demonstrate the presence of non-culturable microorganisms from milk, which has significantly expanded the knowledge of the composition of human milk [32,33].

### 3.1. Archaea

The use of the latest molecular techniques has allowed *Archaea* to be demonstrated in human milk. Previous research has also revealed the DNA of archaea and methanogenic microorganisms in human milk, including *Haloarcula marismortui*, *Halorhabdus utahensis*, and *Halomicrobium mukohataei* [33]. In turn, Togo et al. [55] showed the presence of *Methanobrevibacter smithii* in both colostrum (3 samples) and milk (5 samples). In contrast, *Methanobrevibacter oralis* was cultured from a single sample of human milk [55]. Data about *Archaea* have been largely underestimated in human milk microbiome assessments due to the technical difficulty of the methods used in and their evaluation [56].

### 3.2. Viruses

Data on the human milk virome are limited [57]. Research so far has focused mainly on DNA viruses, leaving aside RNA viruses due to diagnostic difficulties [22].

Available research results indicate the presence of bacteriophages in human milk. Viruses accumulate in the gastrointestinal tract during the first two years of life, indicating environmental acquisition [22]. However, the concentration of bacteriophages in the digestive tract of infants is highest in the first days of life and decreases with age [58]. The analysis conducted by Pannaraj et al. showed that bacteriophages dominated in both infant feces (95.5% ± 3.2%) and mother’s milk (95.2 ± 2.8%). In the same study, a small percentage (4.5 ± 3.2% and 4.8 ± 2.8% of infants and mothers, respectively) in the feces were eukaryotic viruses. The dominant family in infant feces was *Siphoviridae*, while in mother’s milk samples—viruses from the *Myoviridae* family [59]. Duranti et al. showed common bifidophages in infant and maternal fecal samples and human milk. Researchers concluded that bifidophages are transmitted to infants during breastfeeding [57]. In turn, non-phage viral sequences present in human milk samples were identified as members of the families *Papillomaviridae*, *Retroviridae*, and *Herpesviridae* (including the genus *Cytomegalovirus*) [33]. Pannaraj et al. found that a high percentage of bacteriophages that are transmitted from the mother’s milk to the infant’s digestive tract may contribute to shaping the microbiome of the infant’s digestive tract [43].

Human milk may be a carrier of vertical transmission of some viruses, including human immunodeficiency virus (HIV), cytomegalovirus, and human T-cell leukemia [60,61,62]. An intriguing result was the demonstration of the presence of Ebolavirus RNA in the milk of mothers who did not report any symptoms of the disease. It has been suggested that mother’s milk was the source of the Ebola virus to the infant who died from the disease [63]. Transmission of SARS-CoV-2 through human milk has not been demonstrated so far [64,65].

### 3.3. Bacteria

The microbiome of human milk has a diversity of bacterial genera and species. With the development of sequencing methods, the DNA of many non-culturable species has been demonstrated [32,33]. Defining the core composition of the microbiome is extremely difficult, as many factors influence its modulation [34]. The dominant bacteria in breast milk belong to the genera *Streptococcus*, *Staphylococcus*, *Micrococcus*, *Lactococcus*, *Lactobacillus*, and *Bifodobacterium*, but the presence of different species in human milk is variable. [17].

It is also important to estimate the number of bacteria that are present in human milk. Boix-Amorós et al. [27] estimated that an infant may consume approximately 10^7^ to 10^8^ bacterial cells per day through mother’s milk (~800 mL). A study conducted by Jiménez et al. showed that the number of bacteria in human milk (based on 10 samples) ranged from 2.24 to 2.62 log_10_ CFU/mL. However, the ratio of human DNA to microbial DNA was approximately 9:1 [33]. These data show that microbiota make up a significant percentage of human milk, indicating their important role.

Previous studies have identified three predominant phyla (>10%) found in human milk: *Proteobacteria*, *Firmicutes*, and *Bacteroidetes* [33,48,50]. However, the dominant genera in milk from healthy women include *Streptococcus*, *Staphylococcus*, *Lactococcus*, *Ruminococcus*, *Lactobacillus*, *Bifidobacterium*, *Bacterides*, *Faecalibacterium*, *Weisella*, *Leuconostoc*, and *Cutibacterium* [33,48]. The most frequently isolated species are *Staphylococcus epidermidis*, *S. aureus*, *Streptococcus mitis*, *S. salivarius*, *Lactobacillus salivarius*, *L. fermentum*, *L. gasseri*, *L. rhamnosus*, *Bifidobacterium breve*, *B. bifidum*, and *B. longum* [66,67,68,69,70].

Microbiota diversity also depends on the stage of lactation. It was shown that the following genera predominate in colostrum samples: *Weisella*, *Leuconostoc*, *Staphylococcus*, *Streptococcus*, and *Lactococcus*. However, in samples collected during the 6-month feeding period, the dominant species were *Veillonella*, *Leptotrichia*, and *Prevotella* [48] (Figure 2). The higher species diversity in colostrum may be due to the higher concentration of colostrum nutrients. Soto et al. showed that *S. epidermidis* was present in 77.27% of the samples, and the *Streptococcus* genus (dominant species: *S. mitis*, *S. salivarius, and S. parasanguinis*) was present in 60.61% of the tested samples [71]. Recently, a new species isolated from human milk was *Streptococcus lactarius* [69]. In turn, Jiménez et al. showed that *Staphylococcus aureus* was the dominant species in milk samples from women with acute mastitis. However, in women suffering from subacute mastitis, the predominance of *S. epidermidis* has been demonstrated [33]. In turn, the presence of *Enterococcus* spp. in human milk is variable and ranges from 0.5 to 4.0% of the total bacterial DNA [46,48].

Probiotic bacteria have also been identified in human milk. The presence of probiotic strains in human milk has a positive effect on the development of the intestinal microbiota of newborns. It should be emphasized that some strains of lactic acid bacteria (LAB) isolated from human milk have shown the ability to inhibit the growth of pathogenic bacteria. The mechanism of action is based mainly on the production of antimicrobial bacteriocins by LAB, among other things [72,73]. It should be emphasized that *Lactobacillus* bacteria present in milk may inhibit the growth of strains of the following genera: *Pseudomonas*, *Escherichia*, and *Serratia* [74]. The strains with probiotic properties isolated so far included *L. gasseri*, *L. salivarius*, *L. fermentum* (inducing the production of interleukin 10) [75], and *L. rhamnosus* (exhibiting antioxidant and anticancer properties) [76]. Colostrum samples were found to have a higher percentage of *Bifidobacterium* or *Lactobacillus* (76.9% and 48.6%, respectively) compared to mature milk samples [77].

However, Jiménez et al. [33] and Jost et al. [78] also showed the DNA of obligate anaerobic bacteria, i.e., *Bacteroides* spp., and bacteria that synthesize butyrate (*Roseburia* spp., *Eubacteriumrectale*, and *Faecalibacterium prausnitzii*) in human milk. These bacteria are important for proper gut colonization [32,78]. Recently, high-throughput sequencing has revealed the presence of gut-associated, strictly anaerobic microorganisms belonging to the *Clostridiaceae* (*Blautia*, *Clostridium*, *Collinsella*, and *Veillonella* spp.). The presence of specific microorganisms common to the maternal microbiota, human milk, and infant gut microbiota has also been confirmed [38,79,80].

The microbiome of human milk is variable and shaped by many factors. The qualitative and quantitative composition of bacteria in milk is constantly changing [37] and is influenced by many factors. For this reason, this section of the paper provides an overview of the basic human milk microbiome. Characterization of the composition of the microbiome, depending on various factors, is presented later in the manuscript.

### 3.4. Fungi

The presence of fungi in human milk is estimated at approximately 10^5^ CFU/mL [26]. The dominant genera in milk include *Saccharomyces*, *Malassezia*, *Alternaria*, *Rhodotorula*, and *Candida* [26,31]. The presence of fungi of the *Basidiomycota* and *Ascomycota* genera in most milk samples was demonstrated by Jiménez et al. [33]. The tested samples showed the presence of the following genera and species: *Caloceracornea*, *Guepiniopsisbuccina*, *Malasseziaglobosa*, *Podosporaanserina*, *Sordariamacrospora*, *Candida dubliniensis*, *Malasseziarestricta*, *Talaromycesstipitatus*, and *Yarrowialipolytica* [33]. In turn, Boix-Amorós et al. [44] showed that 89% of tested milk samples from healthy women had detectable levels of fungal DNA, with an estimated median load of 3.5 × 10^5^ CFU/mL. Among the samples examined (pyrosequencing), they also showed that the dominant genus was *Malassezia* (44%), followed by *Candida* (19%) and *Saccharomyces* (12%). Additionally, researchers have assessed the impact of ingredients present in milk on the presence of fungi. A positive correlation was found between *Malassezia* spp. and the bacterial load, as well as between this genus and lactose. However, a positive correlation was demonstrated for *Candida* spp. and milk protein [44]. The origin of fungi in human milk is unknown. It should be emphasized that the species isolated in the study by Boix-Amorós et al. [44] are present on human skin, but also in other niches on the body (including *Malassezia*, *Candida*, *Aspergillus*, and *Penicillium*), or isolated from the human intestine (including in. *Candida*, *Malassezia*, *Cladosporium*, or *Debaromyces*) [81,82,83]. Due to its lipophilic nature, the genus *Malassezia* colonizes the seborrheic parts of the skin and is maintained by the use of fatty acids present in sebum [84]. These properties may promote the survival and growth of *Malassezia* spp. in milk, which is characterized by high levels of fat. Boix-Amorós et al. [44] showed that the genera identified among four female populations were *Malassezia*, *Davidiella*, *Sistotrema*, and *Penicillium*. In contrast, the genera *Wallemia* and *Aspergillus* were found only in samples of women from Finland [42]. Further work should indicate the origin of fungi present in milk and their impact on the health of newborns and the development of the microbiome.

### 3.5. Protozoa

There is limited data regarding protozoa in human milk. The first data on the possibility of parasite transmission (*Toxoplasma gondii*) with breast milk were presented by Bonametti et al. [85]. Guého et al. [84] concluded that breastfeeding could be a source of *T. gondii* in the case of a mother who was ill with toxoplasmosis. Jiménez et al. [33] showed the presence of *T. gondii* in 35% of the tested samples and *Giardia intestinalis* in only one milk sample (10%). In contrast, Khamsian et al. [86] showed that out of 300 human milk samples, 1 sample (0.3%) was positive for *T. gondii*.

Chagas disease (infection with the protozoan *Trypanosoma cruzi*) is a major parasitic disease in the Americas and one of the main neglected tropical diseases [87]. To date, only two studies, from 1936 [88] and 1983 [89], have shown the presence of trypomastigotes in the milk of mothers who were in the acute phase of Chagas disease. However, in 1993, a colleague of Prof. Mazza published a letter to the editor [88], in which he corrected the issue of transmission of *T. cruzi* with breast milk. Jörg [90] corrected that the milk collected by Mazza et al. [88] was contaminated with blood, indicating that transmission of *T. cruzi* was via blood (bleeding nipples). Similar results related to the transmission of *T. cruzi* via nipple digestion were presented by Mediana-Lopez [91]. Data on the transmission of *T. cruzi* through breastfeeding in humans are scarce. The data are not up-to-date and are carried out on a small number of patients. Nevertheless, there are suspicions about the possible transmission of *T. cruzi* with breast milk, so this topic should be further analyzed.

Much of the data relate to the presence of antibodies in breast milk that protect the infant from infection, among others: *Giardia lamblia* [92], *Strongyloides stercoralis* [93], *Plasmodium falciparum* [94], or *Onchocerca volvulus* [95].

## 4. Factors Influencing the Microbiome of Human Milk

According to the literature, genetic factors, nutrition of a breastfeeding woman, method of delivery, stage of lactation, time of day, and geographical factors influence the composition of human milk. It is believed that the above-mentioned factors can modulate the microbiota, including the mother’s skin or intestines, as well as the infant’s microbiota. Therefore, maternal, perinatal, environmental, and cultural factors may influence the milk microbiome. Currently, there is no detailed analysis of the human milk microbiome and no detailed information on the impact of factors on the human food microbiome. Knowledge of a given topic and possible modulation of the human milk microbiota could have an impact on the colonization of microorganisms in infants, as well as on the development of the infants’ immune system.

### 4.1. The Influence of Environmental and Cultural Factors on the Microbiome of Human Milk

Geographical location is also among the factors that may influence the composition of the microbiome. Both the area of residence and the presence of urban and rural areas influence the qualitative and quantitative composition of human milk.

A higher number of bacteria was found in the milk of women from rural areas. Taghizadeh et al. showed that the median number of *Lactobacillus* bacteria was higher in women living in rural areas than in women living in urban areas [96]. Also, Sinkiewicz et al. showed that the milk of women living in rural areas is characterized by a higher number of lactobacilli (1.3 × 10^3^ CFU/mL) compared to women living in cities (3.0 × 10^2^ CFU/mL). Moreover, in women living in rural areas, they showed the presence of *L. reuteri* in 14.0% of the samples, while in women from urban areas, the same species was found in 15.0% of the samples [97].

Geographical location also influences the composition of the microbiome. For example, higher numbers of lactobacilli have been found in women in Israel, South Africa, Japan, and South Korea [97]. In turn, González et al. showed that the most frequently isolated genera from mother’s milk among Mozambican women were *Staphylococcus* (96.4%), *Streptococcus* (92.7%), and *Lactobacillus* (56.4%). However, HIV RNA was detected in 24.0% of the milk samples. Researchers have also observed higher bacterial diversity and the prevalence of *Lactobacillus* spp. in milk samples from HIV-positive women [98]. In turn, a study conducted by Urbaniak et al. using sequencing (Ion Torrent) confirmed that the milk of healthy women living in Canada was dominated by the genera *Actinobacter*, *Stenotrophomonas*, *Pseudomonas*, *Streptococcus*, and *Staphylococcus*. However, the milk of Caucasian women living in Canada was dominated by *Pseudomonas*, *Streptococcus*, and *Lactobacillus* [99]. Dave et al. showed that Streptococcus was the dominant genus in the milk of Mexican-American women [100]. However, Boix-Amorós et al. showed that the milk microbiota among Spanish women was dominated by the following genera: *Staphylococcus*, *Pseudomonas*, *Streptococcus*, and *Acinetobacter*. Importantly, researchers have shown that the median bacteria in human milk is approximately 10^6^ CFU/mL, suggesting that breastfed infants consume approximately 7–8 million bacterial cells per day [27]. A study by Gonzalez et al. involving lactating women from Guatemala showed that ten different species of the *Staphylococcus* genus were associated with early lactation, including *S. hominis*, *S. epidermidis*, and *S. hyicus*. The dominant species of the *Streptococcus* genus identified were *S. mitis*, *S. parasanguinis*, *S. peroris*, *S. pneumoniae*, *S. pseudopneumoniae*, and *S. salivarius*. Other species identified in milk during early lactation included *Corynebacterium tuberculostearicum*, *C. jeikeium*, *L. gasseri*, *Acinetobacter johnsonii*, *Kocuria palustris*, and *Janthinobacterium agaricidamnosum*. In turn, bacteria associated with the late phase of lactation among breast feeding women from Guatemala included species of the genera *Staphylococcus* and *Streptococcus* (which were associated with the early phase of lactation) and species such as *Sphingobium yanoikuyae*, *Pseudomonas putida*, *Stenotrophomonas maltophilia*, *Ottowia beijingensis*, and *Comamonas testosteroni* [101].

The analysis conducted by Kumar et al. showed the highest level of *Bacteroidetes* in Spanish women (natural childbirth) compared to women from other countries (Finland, South Africa, and China). However, women from South Africa showed a significantly higher abundance of Proteobacteria compared to other samples. The samples from Finnish women had higher levels of Firmicutes and lower levels of Proteobacteria when compared to the samples from other countries (*p* = 0.004). The highest levels of *Actinobacteria* were observed among mothers from China who gave birth vaginally. At the genus level, Chinese women had higher levels of *Streptococcus*, and Spanish women had higher levels of *Cutibacterium* and *Pseudomonas*. *Lactobacillaceae* was found exceptionally in samples from Finland, *Bifidobacteriace* was found only in South African women, and *Enterococcaceae* was found in samples from all countries except China [102]. However, Davé et al. showed that the predominant genera in milk from Mexican-American women were *Streptococcus*, *Staphylococcus*, *Xanthomonadaceae*, and *Sediminibacterium* [100].

Lackey et al. showed that *Staphylococcus* and *Streptococcus* bacteria were present among all analyzed milk samples (regardless of the women’s place of residence). However, the genus *Rhizobium* was detected in the milk of women in a rural community in Ethiopia. In turn, among the samples of milk from women from Peru, it was shown that 50% of the bacterial community was the *Streptococcus* genus, and this level was significantly higher compared to the other examined women’s communities (from Africa and the United States). Interestingly, milk from women from the Washington site had higher levels of *Dyella* spp. [103].

Data on microbiome diversity depending on geographical location are presented in Table 1. A study by Boix-Amorós et al. [42] showed that geographical location did not affect the amount of fungi in human milk, but there were differences in qualitative assessment. Researchers confirmed that samples from South African women had significantly higher levels of *Ascomycota* spp. and lower levels of *Basidiomycota* spp. and *Malassezia* spp. than in other population studies. In turn, milk samples from Chinese women showed lower levels of fungi from the genus *Penicillium* and *Rhodotorula*, while the genus *Saccharomyces* was more abundant in milk samples from Spanish and Finnish women compared to other geographical locations [42].

### 4.2. The Influence of Maternal Factors on the Microbiome of Human Milk

Maternal factors affecting the human milk microbiome include age, body mass index (BMI), taking medications, type of diet, supplementation, physical activity, lifestyle, stimulants, and diseases accompanying breastfeeding women (Table 2) [7,117,118,119].

The stage of lactation has a significant impact on the formation of milk microbiota. Cabrera-Rubio et al. [48] compared the microbiota of colostrum and mature milk collected 1 and 6 months after delivery. Bacteria from the genera *Firmicutes*, *Weissella*, and *Leuconostoc* (*Lactobacillales*) predominated in colostrum, followed by *Staphylococcus*, *Streptococcus*, and *Lactococcus*. These genera were also abundant in samples collected at a later stage, but the numbers of the genera *Veillonella* (*phylum Firmicutes*), *Leptotrichia* (*Fusobacteria*), and *Prevotella* (*Bacteroidetes*) increased [48]. However, there are studies that do not confirm this relationship. Li et al. showed that the milk microbiome at 3.6 months and above 6 months of lactation does not show diversity [7].

The mother’s health shapes the microbiota of human milk, in particular the woman’s obesity and diseases accompanying breastfeeding women (e.g., celiac disease, allergy). Maternal obesity reduces microbial diversity, the number of *Bifidobacterium* spp. and *Lactobacillus* and increases the number of *Staphylococcus* [120]. Other results were presented by Moosavi et al., indicating that BMI and maternal cigarette smoking do not affect the human milk microbiome [45]. Similar results were obtained by Li et al. [121].

There are limited data on the impact of infection and comorbidities of breastfeeding women on the microbiota of human milk. Currently, the impact of celiac disease, HIV, ormastitis on the composition of human milk, including the microbiome, has been described [98,107]. Mastitis is a state of dysbiosis in which the number of bacteria increases to one million CFU/mL. Research by scientists from Spain showed that milk samples from 1849 women suffering from acute, subacute, or subclinical mastitis were 60% dominated by *S. epidermis*. In the remaining 40% of samples, at least one of the dominant species was a species of the genus *Streptococcus* (often from the *salivarius* or *mitis* group), as well as *S. aureus* and *S. epidermis* [122].

Tuominem et al. did not detect differences in the composition of human milk microbiota related to HPV infection. However, it is important to note that this study included a small study group [123]. Similar results were obtained by Bender et al. in relation to HIV [114].

Taking medications (including antibiotics), supplements, and chemotherapy may have a significant impact on the human milk microbiome. A link between the occurrence of microorganisms in human food and intrapartum exposure to antibiotics in milk a month after delivery has been demonstrated. Lower numbers of lactobacilli and *Bifidobacterium* spp. were observed [124]. Subsequent studies show an increased presence of bacteria of the genus *Acinetobacter*, *Xanthomonadacea*, as well as a reduced presence of *Bifidobacterium*, *Staphylococcus*, and *Eubacterium* in the milk of women undergoing chemotherapy. Supplementation during pregnancy and lactation is extremely important for both the woman and the child. A positive correlation was found between vitamin C intake during pregnancy and the number of *Staphylococcus* spp., as well as the impact of maternal vitamin B1 and B2 intake on the milk microbiome [125].

The impact of a breastfeeding woman’s diet on the human milk microbiome has not been thoroughly investigated. Few scientific studies suggest that diet affects not only the intestines but also the composition of human milk, including the microbiome. A correlation has been noted between the intake of saturated (SFA) and monounsaturated fatty acids (MUFA), carbohydrates, protein, and the occurrence of microorganisms in milk [126]. Cortes et al. associated carbohydrate intake with the occurrence of *Staphylococcus* spp. and *Bifidobacterium* spp. in human milk, while the genus *Streptococcus* was associated with the intake of *n*-3 PUFA [EPA and docosapentaenoic acid (22:5ω-3)] [125]. Scientists [104,120] believe that the mother’s diet during pregnancy may have a greater impact on the human food microbiome compared to the diet during lactation. Williams [120] obtained dietary intake data and linked it to milk microbiome data obtained from 21 lactating women at the same time. Few associations were found between dietary intake variables and the relative abundance of bacteria in milk. However, when dietary and microbiome data are averaged over the entire observation period, a myriad of significant associations of phyla or phylum, respectively, with specific nutrients (e.g., negative association between *Corynebacteria* and SFA and MUFA) and macronutrients (e.g., reported carbohydrate intake and *Firmicutes*) can be found [120]. In turn, Drago et al. showed no effect of diet on the microbiome of human milk [104].

**Table 2:** Diversity of the breast milk microbiome depending on maternal factors.

Maternal Factor	Number of Women; Residence	Microbiota Analysis Method	Microbiota Diversity	References
Overweight/obesity of a breastfeeding woman	normal-weight (*n* = 8) and obese (*n* = 10) mothers	16S rRNA gene sequencing	● higher number of *Staphylococcus* spp., but lower number of *Bifidobacterium* spp. in obese women compared to normal-weight women.	Cabrera et al. [48]
Antibiotic therapy	160 women (40.62% had received antibiotherapy)	16S rRNA gene sequencing	● lower number of the genera *Lactobacillus*, *Bifidobacterium*, *Eubacterium*, and *Staphylococcus* in women receiving antibiotic therapy compared to women who did not use antibiotics during pregnancy and lactation.	Soto et al. [71]
Chemotherapy	8 healthy women/8 women undergoing the ABVD chemotherapy	16S sequencing and the metabolome by gas chromatography–mass spectrometry	● lower number of *Lactobacillus*, *Bifidobacterium*, *Eubacterium*, *Staphylococcus*, and *Cloacibacterium* spp. In women undergoing the ABVD chemotherapy compared to healthy women.	Urbaniak et al. [99]
Celiac disease	12 healthy mothers/12 mothers with CD	PCR; MOLECULAR MASS	● lower number of *Bifidobacterium* spp. and *Bacteroidesfragilis* in women with CD compared to healthy women.	Olivares et. al. [127]
HIV	121 women (23% with HIV)	Culture	● higher bacterial diversity and higher number of *Lactobacillus* spp. In milk samples with HIV RNA than in samples without it.	Gonzalez et al. [98]
Lactation phases	18 women/colostrum and mature milk	16S rRNA gene sequencing	● colostrum samples were dominated by *Weisella*, *Leuconostoc*, *Staphylococcus*, *Streptococcus*, and *Lactococcus*; ● colostrum showed different patterns of bacterial diversity compared to 6-month-old milk samples; ● lower number of *Bifidobacterium* spp. in breast milk 6-month-old milk samples were related to higher maternal BMI.	Cabrera et al. [48]
Antibiotics/Caesarean section/diet	120 women divided into: ● Cluster I (high intake of plant protein, fiber, and carbohydrates) and Cluster II (high intake of animal protein and lipids) ● Caesarean section/natural birth Taking antibiotics/not taking antibiotics	16S rRNA gene sequencing	● in group II/section C/exposure to antibiotics, a lower number of *Lactobacillus*, *Bacteroides* and *Sediminibacterium* genera was observed compared to other groups.	Cortes-Maicas [125]
Probiotics (Lactobacillus Salivarius CECT5713 and Lactobacillus Fermentum CECT5716 strains)	Women with (*n* = 23) and without (*n* = 8) symptoms of mastitis received three daily doses (10^9^ CFU) of *Lactobacillus salivarius* PS2 for 21 days.	PCR	● supplemented strains were detected in milk; ● reducing the number of bacteria in milk	Espinaso et al. [128]
Mastitis	50 breast milk samples, including 16 subacute mastitis (SAM), 16 acute mastitis (AM) and 18 healthy control samples	16S rRNA gene sequencing	● higher number of *Aeromonas*, *Staphylococcus*, *Ralstonia*, *Klebsiella*, *Serratia*, *Enterococcus*, and *Pseudomonas* in SAM and AM samples; ● lower number of *Acinetobacter*, *Ruminococcus*, *Clostridium*, *Faecalibacterium*, and *Eubacterium* in SAM and AM samples.	Patel et al. [107]
Diet	113 milk samples	PCR	● 1 g increase in fiber content in cereals was associated with reduced incidence of *Fusobacteria* and *Streptococcus* and an increase in *Acinebacterium*; ● trans fats showed a positive relationship with the occurrence of *Staphylococcus* and *Gemella*; ● A 1 g increase in monounsaturated fat intake was associated with an increased incidence of *Acinetobacter* and *Gemella;* ● negative associations were observed between the consumption of polyunsaturated fats and the incidence of *Acinetobacter.*	LeMay-Nedjelski et al. [129]
Lactation phase	47 breastfeeding women	MALDI-TOF-MS	● total number of bacteria higher in colostrum than mature milk.	Damaceno et al. [106]
Mastitis	20 women (10 healthy, 10 mastitis)	MALDI-TOF-MS	● high absolute abundance of *S. aureus* in women with acute mastitis and *S. epidermidis* in women with subacute mastitis.	Jiménez et al. [33]
A type of breast pumping	393 women	16S rRNA gene sequencing	● higher content of *Gemellaceae*, *Vogesella*, and *Nocardioides* with manually expressed milk, higher relative abundance of *Enterobacteriaceae* and *Pseudomonas* in milk expressed by a breast pump.	Moossavi et al. [45]
BMI	21 women	16S rRNA gene sequencing	● women with higher BMI had higher abundance of *Granulicatella* and lower relative abundance of *Bacteroides.*	Williams et al. [120]
Lactation phases	50 women	16S rRNA gene sequencing	● higher number of anaerobic bacteria in mature milk compared to colostrum.	Drago et al. [104]
Taking antibiotics	20 women	16S rRNA gene sequencing	● milk from women taking 25% IAP (ampicillin 5) showed a lower total bacterial count (10^4^–10^6^ CFU/mL).	Solis et al. [68]
Lactation phases	22 women	Culture	● higher number of *Enterococcus*, *Lactobacillus* and *Streptococcus* spp. in mature milk	Moles et al. [130]

rRNA—ribosomal ribonucleic acid; CD—celiac disease; PCR—polymerase chain reaction; HIV—human immunodeficiency virus; MALDI-TOF-MS—matrix-assisted laser desorption/ionization time of flight mass spectrometry; ABVD—chemotherapy drug combination that includes doxorubicin, bleomycin, vinblastine, and dacarbazine; SAM—subacute mastitis; AM—acute mastitis; CFU—colony-forming unit; BMI—body mass index.

### 4.3. Perinatal Factors

Scientific reports show that, from the neonatal period to infancy, the method of delivery is an important factor influencing the composition of the intestinal microbiota (Table 3). Moreover, research conducted on 596 full-term children showed that the method of delivery is the most important factor influencing the composition of children’s intestinal microbiota [131]. Newborns born vaginally acquire bacteria from the mother’s birth canal, mainly species of the genus *Lactobacillus* and *Prevotella*. However, children born by cesarean section acquire bacteria that resemble the mother’s skin microbiome, such as *Staphylococcus* spp. [132,133]. Babies born by cesarean section have lower levels of anaerobic bacteria, such as *Bacteroides* spp. and *Bifidobacterium* spp. and are also colonized by such bacteria as *Clostridium*, *Cutibacterium*, and *Corynebacterium* [134]. Cesarean section delivery has also been associated with lower abundance and diversity of the *Actinobacteria* and *Bacteroidetes phyla*, as well as greater abundance and diversity of the *Firmicutes phylum* from birth to 3 months of age [135]. The conclusions of the systematic review seem interesting, indicating that the method of delivery is most important for the diversity and colonization pattern of the intestinal microbiome in the first three months of a child’s life. However, the method of delivery has a smaller impact on the colonization and diversity of *Bifidobacteria*, *Bacteroides*, *Clostridium*, and *Lactobacillus genera* from 6 to 12 months of age [136].

Another important factor influencing a child’s intestinal microbiome is the duration of pregnancy. Research analysis indicates that full-term children have a different composition of intestinal microbiota than premature babies [137]. Research indicates that at all stages of lactation, a group of premature babies has significantly lower levels of *Bifidobacterium* spp. than a group of full-term babies [76].

Intestinal colonization in premature babies is influenced by many factors that cause disturbances in the intestinal ecosystem or dysbiosis—including necrotizing enterocolitis (NEC), which is the main cause of mortality in premature infants [138]. Interesting studies on a group of 29 premature infants born between 28 and 32 weeks of pregnancy indicate a higher number of *Clostridiates* and a lower number of *Enterobacterium* among female infants compared to male infants [139]. Subsequent studies conducted on premature babies show low species diversity. In this study, *Bacilli*, *Proteobacteria*, and *Clostridium* were the most abundant and accounted for 87%, and *Actinobacteria* and *Bacteroidia* 6.5% and 5.1%, respectively [140]. The intestinal microbiome of premature infants consists mainly of bacteria of the genera *Enterococcus*, *Staphylococcus*, *Streptococcus*, *Citrobacter*, *Enterobacter*, *Escherichia* (mainly *E. coli*), *Klebsiella*, *Raoultella*, *Serratia*, and *Shigella*, and the *Enterobacteriaceae* order. There are also genera such as *Bacteroides*, *Clostridium*, and *Veillonella*. *Bifidobacterium* and *Lactobacillus* (intestinal bacteria), whose function is to protect the intestines against pathogens, are present in premature infants only two months after birth. Moreover, in prematurely born children, in the first six weeks there is a decrease in the number of bacteria of the genera *Staphylococcus*, *Shigella*, *Escherichia*, and *Prevotella*, and an increase in bacteria such as *Enterococcus*, *Streptococcus*, *Enterobacteriaceae*, and *Veillonella*. Studies also indicate that the occurrence of NEC is associated with a more frequent presence of *Enterobacteriaceae*, *Clostridium* spp., and coagulase-negative staphylococci (CNS). However, the presence of *Enterococcus faecalis* is associated with a reduced risk of NEC [141]. Willoughby et al. indicated a relationship between the occurrence of NEC and the presence of bacteria such as *E. coli*, *Klebsiella* spp., *Enterobacter* spp., *Clostridium butyricum*, *Clostridium perfringens*, *Salmonella* spp., *Pseudomonas aeruginosa*, *Clostridioides difficile*, CNS, *S. aureus*, *C. glabrata*, coronavirus, enterovirus, and rotavirus [142]. Another study conducted on a group of 369 premature infants showed that the presence of *Klebsiella* spp. and *Clostridium* spp. was strongly associated with an increased risk of NEC in children [143].

Postnatal exposure of the child to various environments during early intestinal development influences the colonization of the gastrointestinal tract and its immune system. Studies conducted from the 3rd to the 39th day of life on 58 premature babies staying in the Neonatal Intensive Care Unit (NICU) showed that approximately 92% of all bacteria in the stool are *Proteobacteria* (54%), *Bacillus* (19.3%), and *Clostridia* (18.4%) [144]. Interesting research was carried out in the Intensive Care Unit (ICU), where samples of bedding, frames, and furniture were taken after cleaning twice with a 500 ppm free chlorine solution. The results of sample analysis showed the presence of bacteria such as *Enterococcus* spp., *S. aureus*, *Klebsiella* spp., *Acinetobacter* spp., *P. aeruginosa*, and other *Enterobacteriaceae*. These microorganisms also occur on the surface of the NICU and are the most common cause of hospital infections [145]. Another study assessing 16 NICU room surfaces showed that the majority of microorganisms were associated with the skin—over 50% (*Corynebacterium* spp.)—mouth (*Streptococcus* spp.), or nose (*Staphylococcus* spp.). Interestingly, the floor in front of the infant isolation room had the highest microbial density compared to any other NICU environment [146].

According to the literature, the composition of the human milk microbiome is also influenced by parity. In their research, Kim et al. observed a greater presence of *Staphylococcus* spp. and *Haemophilus* spp. in bacterial samples from multiparous women compared to samples from primiparous women [147].

Scientific research shows that the vaginal microbiome changes with gestational age [148]. Moreover, subsequent research analyzes suggest that when the microbiological balance in the vaginal ecosystem is disturbed, the risk of premature birth increases [135]. Research conducted as part of the Human Microbiome Project (iHMP) of the National Institute of Health showed that premature birth was associated with low numbers of *Lactobacillus* spp. bacteria in the vagina, especially among African American women. These studies also showed a positive correlation of four taxa: *S. amnii*, *BVAB1*, *Prevotella* cluster 2, and TM7-H1—with the occurrence of preterm birth, which may be important in predicting the risk of preterm birth [149]. La Rosa et al. observed that the intestinal microbiota of premature infants staying in a strictly controlled microbiological environment changes from *Bacilli* to *Gammaproteobacterial* to *Clostridia*. Moreover, when prematurely born children approach 33–36 weeks of post-conceptional age, their intestines are well colonized by anaerobes [144].

An analysis of intrapartum antibiotic therapy showed a delay in the maturation of microbial activity from 6 to 12 months after delivery [150]. An increase in the number of *Enterobacteria* in infants treated with antibiotics and a reduction in the number of *Bacteroides* and *Atopobium* clusters in children born to mothers treated with antibiotics during pregnancy or lactation were observed [151]. In their research, Hermansson et al. noticed the absence of *Bifidobacterium* spp. bacteria in the milk of women who received intrapartum antibiotic therapy [124]. The results of this study may be important because reduced *Bifidobacterium* spp. in early infancy increases the risk of atopy and obesity [30]. Intrapartum exposure to antibiotics also influences the development of NEC [135], as well as the occurrence of infantile colic [152].

The method of feeding plays an important role in shaping the microbiota of human milk. According to the literature, direct breastfeeding has a positive effect on the absorption of bacterial microbiota from the child’s oral cavity, while feeding the child with expressed human milk increases the risk of acquiring bacteria from the environment [75]. Moossavi et al. observed a significantly higher prevalence of *Bifidobacterium* spp. in directly breastfed children than in children fed with expressed human milk. Furthermore, *Gemellaceae*, *Vogesella*, and *Nocardiosis* were more abundant in direct breastfeeding, while *Enterobacteriaceae* and *Pseudomonas* spp. Were relatively more abundant in indirect breastfeeding [45]. Many scientific studies have indicated differences in the composition of the intestinal microbiota between breastfed and formula-fed children. Through breastfeeding, the child acquires a large amount of *Bifidobacterium* spp., which constitutes approximately 90% of the total intestinal microbiome in the first year of the child’s life [153]. This is confirmed by subsequent studies indicating the predominance of *Bifidobacteria* in the feces of exclusively breastfed infants, while *Enterococcus* spp. and *Clostridium* spp. predominated in formula-fed children [28,154,155]. In addition, children fed with formula milk acquire more microorganisms such as *E. coli*, *C. difficile*, *Bacteroides*, *Firmicutes*, and *Lactobacilli* [156,157]. Zimmermann et al., reviewing the factors influencing the composition of the intestinal microbiome in the first year of life, indicate that in breastfed children, a greater number of *Staphylococcus* and *Streptococcus* species are observed, while in children fed with formula milk: *Bacteroides*, *Clostridium*, *Enterobacteriaceae*, *Escherichia*, *Klebsiella*, *Enterococcus*, and *Lachnospiraceae*, with slower colonization by *Bifidobacterium* spp. [141]. Research conducted on a group of 28 premature infants indicates that a greater diversity of the intestinal microbiome and a significantly higher number of *Clostridiales* and *Lactobacillus* bacteria are observed in premature infants fed with mother’s milk than in premature infants fed with modified milk and/or donor milk [139]. However, Korpela et al. found that the intestinal microbiome in breastfed premature infants develops in four phases. The first phase peaks between 25 and 30 weeks of post-conceptional age with the dominance of *Staphylococcus* spp. The second phase is dominated by *Enterococcus* spp., and the peak occurs between 30 and 35 weeks of post-conceptional age; the third phase, *Enterobacter* spp., and the peak at 35 weeks. The highest number of *Bifidobacterium* spp. was observed in the fourth phase, recorded after the 30th week of post-conceptional age. The third phase, dominated by *Enterococcus* spp. bacteria, was observed only in extremely premature infants and seemed to delay the succession of microbiota [158].

**Table 3:** Diversity of the gut microbiome of newborns and infants depending on perinatal factors.

Perinatal Factor	Number of Study Group	Microbiota Analysis Method	Microbiota Diversity	References
Method of delivery	46 newborns (*n* = 23 born vaginally, *n* = 23 born by cesarean section)	PCR	● lower numbers of *Bifidobacterium* and *Bacteroides* in infants born by cesarean section compared to infants born vaginally.	Biasucci et al. [159]
Method of delivery	116 newborns (*n* = 99 born vaginally, *n* = 17 born by cesarean section)	PCR and culture	● higher abundance of *Escherichia coli* in newborns born vaginally; ● in newborns born by cesarean section, a higher number of enterobacteria, i.e., *Klebsiella* and *Enterobacter*; ● *Bacteroides* colonization delayed up to 1 year in children born by cesarean section.	Adlerberth et al. [160]
Method of delivery	64 newborns (*n* = 34 born vaginally, *n* = 30 born by cesarean section)	culture	● *Clostridium perfringens* colonization rate statistically higher in the group of children born by cesarean section than in the group of children born vaginally in the first month of life (57% vs. 17%); ● colonization rates of *Bifidobacterium*-like and *Lactobacillus*-like bacteria reached the colonization rate of vaginally delivered children after 1 month and 10 days, respectively; ● *Bacteroides* colonization was not detected in any stool sample from infancy in children born by cesarean section until the age of 2 months.	Minna-Maija et al. [161]
Method of delivery	10 newborns (*n* = 4 born vaginally, *n* = 6 born by cesarean section)	multiplexed pyrosequencing of the 16S rRNA gene	● *Lactobacillus* dominance in infants after vaginal delivery; ● *Prevotella* or *Sneathia* spp., in infants after cesarean section, the dominance of *Staphylococcus*, *Corynebacterium*, and *Cutibacterium* spp.	Dominguez-Bello et al. [132]
Duration of pregnancy	29 premature babies (28 and 32 weeks of pregnancy)	16S rRNA gene sequencing	● the most numerous phylum *Proteobacteria*; ● current shift patterns: increase in *Clostridium* and *Bacteroides* and decrease over time in early life; ● higher numbers of *Clostridiates* and lower numbers of *Enterobacterium* in girls than in boys.	Cong et al. [139]
Duration of pregnancy	120 children (*n* = 25 children born between 23 and 25 weeks of pregnancy; *n* = 22, 26–27 weeks of pregnancy; *n* = 11, 28–29 weeks of pregnancy, *n* = 11, 30–31 weeks of pregnancy, *n* = 18, 32–33 weeks of pregnancy; *n* = 8, 34–35 weeks of pregnancy; *n* = 25, 37 weeks of pregnancy and above	16S rRNA gene sequence	● in premature infants, the most numerous phylum of *Bacilli*, *Proteobacteria*, *Clostridia* (87%); ● *Actinobacteria* and *Bacteroidia* 6.5% and 5.1%, respectively.	Grier et al. [140]
Duration of pregnancy	58 children (*n* = 15 children born < 26 weeks of gestation; *n* = 20, 26–28 weeks of pregnancy; *n* = 23, >28 weeks of pregnancy	16S rRNA gene pyrosequencing	● the intestinal microbiota of premature infants changes from *Bacilli* to *Gammaproteobacteria* to *Clostridia.*	La Rosa et al. [144]
Duration of pregnancy	40 children (*n* = 27 children born between 24 and 32 weeks of pregnancy; *n* = 13 children born between 37 and 41 weeks of pregnancy)	16S rRNA gene sequence	● in premature babies in the first months of life, a lower number of the *Bacteroidaceae* family and a higher number of *Lactobacillaceae* compared to full-term children.	Arboleya et al. [162]
Duration of pregnancy	41 children (*n* = 21 children born between 30 and 35 weeks of pregnancy; *n* = 20 children born between 38 and 41 weeks of pregnancy)	qPCR	● in premature infants, increased number of facultative anaerobes *Enterobacteriaceae*, *Enterococcaceae* and *Lactobacillus* (including *Weissella*); ● in premature babies, reduced numbers of anaerobic bacteria, including *Bifidobacterium*, *Bacteroides*, and *Atopobium*,	Arboleya et al. [163]
Duration of pregnancy	29 premature babies (born between 27 and 29 weeks of pregnancy)	16S rRNA gene sequence	● increase in the number of operational taxonomic units by 0.45 units/week—with staphylococci being the main group; ● little represented bacteria of the *Bifidobacterium* genus.	Jacquot et al. [164]
Duration of pregnancy	29 children born between 24 and 37 weeks of pregnancy	16S rRNA gene sequence, PCR	● dominant bacteria in premature babies: *Escherichia coli*, *Enterococcus* spp. and *Klebsiella pneumoniae*	Schwiertz et al. [165]
NEC	32 children	16S rRNA gene sequence	●Firmicutes and Proteobacteria predominate in children with NEC; ● no *Cutibacterium* spp. present in children with NEC.	Morrow et al. [166]
NEC	122 children	16S rRNA gene sequence	● NEC in very low birth weight infants preceded by an increased abundance of *Gammaproteobacteria* and a deficiency of anaerobic bacteria (especially *Negativicutes*).	Warner et al. [167]
NEC	369 children	16S rRNA gene sequence	● presence genus of *Clostridium* and *Klebsiella* in children with NEC.	Sim et al. [143]
NICU	58 children	16S rRNA gene pyrosequencing	*●* presence of *Proteobacteria* (54%), *Bacillus* (19.3%) and *Clostridia* (18.4%).	La Rosa et al. [144]
NICU	16 private-style NICU rooms	16S rRNA gene sequence, PCR	● over 50% of microorganisms were associated with the skin (*Corynebacterium*), then the oral cavity (*Streptococcus*) and the nose (*Staphylococcus*).	Brooks et al. [146]
Antibiotic therapy	606 children	16S rRNA gene sequence	● increase in the number of *Enterobacter* in infants treated with antibiotics; ● reduction in the number of *Bacteroides* and *Atopobium* clusters in children of mothers using antibiotic therapy.	Fallani et al. [151]
Antibiotic therapy	61 women	16S rRNA gene sequence	● lack of *Bifidobacterium* spp. bacteria in the milk of women who received intrapartum antibiotic therapy.	Hermansson [124]
Reproduction	22 women (16 primiparous, 6 multiparous)	16S rRNA gene sequence, PCR	● higher numbers of *Staphylococcus*, *Collinsella* and *Haemophilus* in multiparous women.	Kim et al. [147]
Feeding method	393 women	PCR	● higher numbers of *Bifidobacterium* spp. in children fed directly by breast milk compared to children fed with expressed mother’s milk; ● in children who are directly breastfed, a relatively higher abundance of *Gemellaceae*, *Vogesella* and *Nocardioides*; ● in children fed with expressed breast milk, a relatively higher number of *Enterobacteriaceae* and *Pseudomonas.*	Moossavi [45]
Feeding method	1032 children	16S rRNA gene sequence, PCR	● higher numbers of *E coli*, *C difficile*, *Bacteroides* and *Lactobacillus* in children fed with formula milk.	Penders et al. [156]
Feeding method	684 children	16S rRNA gene sequence	● in the first 6 months of life, higher numbers of *Bacteroidetes* and *Firmicutes* in formula-fed children.	Ho et al. [157]
Feeding method	98 dyads (mother-child)	culture	● in children fed with formula milk, lower numbers of *Bifidobacterium* and higher numbers of *Clostridium* and *Enterobacteriaceae* (*E. coli*); ● in 4-month-old exclusively breastfed children, a higher abundance of taxa used as probiotics, such as *L. johnsonii/L.gasseri*, *L. paracasei/L. casei* and *B. longum*; ● in 4-month-old children fed with formula milk, higher numbers of *Clostridioides difficile*, *Granulicatella adiacens*, *Citrobacter* spp., *Enterobacter cloacae*, and *Bilophila wadsworthia.*	Bäckhed et al. [155]
Feeding method	29 premature babies (28 and 32 weeks of pregnancy)	16S rRNA gene sequencing	● significantly higher abundance of *Clostridiales* and *Lactobacillales* in premature babies fed with human milk compared to children fed with formula.	Cong et al. [139]
Feeding method	45 premature babies	16S rRNA gene sequencing	● the development of the microbiota took place in four phases, with the dominance of *Staphylococcus*, *Enterococcus*, *Enterobacter* and finally *Bifidobacterium.*	Korpela et al. [158]

DNA—deoxyribonucleic acid; RNA—ribosomalribonucleic acid; PCR—polymerase chain reaction; HIV—human immunodeficiency virus, NEC—necrotizing enterocolitis; NICU—neonatal intensive care unit.

## 5. Composition of Human Milk and the Microbiome

There are several studies in the scientific literature explaining the relationship between the composition of human milk and the microbiome of human milk. One of the few studies at the moment is the analysis by Moosavi et al., in which the general profile of fatty acids in milk was significantly related to the composition of the milk microbiota. It has been shown that fatty acids [22:6n3 (docosahexaenoic acid), 22:5n3, 20:5n3, 17:0, 18:0] and oligosaccharides (fucosyl-lacto-N-hexaose, lacto-N-hexaose, lacto-N -fucopentaose I) were associated with microbiota diversity [45]. Several significant associations were observed between individual HMOs and the microbiota. Among mothers using expressed milk, the prevalence of *Bifidobacterium* spp. was associated with a lower disialyl-lacto-N-hexaose content [45]. Kumar et al. described the association of lipid profile with microbiota. In their study, monounsaturated fatty acids (MUFA) in triacylglycerols (TAG) were negatively correlated with Proteobacteria (r = −0.43, *p* < 0.05). Additionally, over 90% of women in the study were positively associated with MUFA and polyunsaturated fatty acids (PUFA) in TAG, while saturated fatty acids (SAFA) in TAG were negatively associated with *Streptococcus* and *Acinetobacter* genera. In turn, in the case of phospholipids, the genus *Lactobacillus* was negatively associated with the genus MUFA (r = −0.23, *p* = 0.04), and *n*-3 PUFA was negatively associated with the genus *Bifidobacterium* (r = −0.26, *p* = 0.03) [102]. Opposite results suggesting a lack of relationship between the occurrence of oligosaccharides in milk and the food microbiome were obtained by Moossavi et al. [45].

## 6. Microbiota Modulation—During Pregnancy and after Childbirth

Scientific research shows that colonization of the intestines with microorganisms takes place at several stages. According to the literature, the first stage probably begins in the prenatal period and ends several months or even several years after birth. These reports question the dogma of the “sterile” fetus [135]. Currently, the issue of intestinal colonization is still very controversial. There is no consensus among scientists dealing with microbiology and the prenatal/perinatal period. Numerous scientific studies have indicated the presence of microorganisms in fetal tissues [36,168,169], although the latest studies deny this [170]. The results of microbiome analysis conducted using metagenomic shotgun sequencing techniques indicate the presence of commensal bacteria in the uterus, placenta, amniotic fluid, and meconium [46,171,172,173,174,175,176,177].

The studies presented in Table 4 indicate that microbial exchange occurs from mother to fetus, confirming that the fetal environment is not sterile. The study results also indicate the importance of the mother’s microbial environment for the infant’s developing immune system [25]. The mother’s oral microbiota is an important prenatal factor influencing the development of the child’s intestinal microbiome [171,178]. The intestinal microbiome of newborns is also influenced by the duration of pregnancy and the method of delivery, which is described in detail in Section 4.3.

**Table 4:** Diversity of the microbiome from the placenta, amniotic fluid, meconium, and cord blood.

Type of Material Collected	Number of Women; Residence	Microbiota Analysis Method	Microbiota Diversity	References
Placenta	320 women	16S rRNA gene sequencing	● nonpathogenic commensal microbiota from the *Firmicutes*, *Tenericutes*, *Proteobacteria*, *Bacteroidetes*, and *Fusobacteria* phyla.	Aagaard et al. [171]
Placenta	37 overweight and obese women	16S rRNA gene sequencing	● in the *Firmicutes* phylum, the dominant genera are *Streptococcus*, *Lactobacillus*, and *Veillonella*; ● in the *Proteobacteria* phylum, the dominant genera are *Pseudomonas*, *Haemophilus*, and *Acinetobacter.*	Gomez-Arango et al. [172]
Placenta	1391 women	16S rRNA gene sequencing	● the dominant species in fetal membranes are *Lactobacillus iners*, *Gardnerella vaalis*, and *Sneathia sanguinegens*; ● *Acinetobacter* spp. And *Enterobacteriaceae* predominant species in placental tissues.	Doyle et al. [173]
Placenta	64 women	16S rRNA gene sequencing	● three species of *Candida* fungi have been identified: *C. albicans*, *C. tropicalis*, and *C. glabrata* ● 30 species of bacteria were identified; *Escherichia coli* belonging to the *Enterobacteriaceae* family constituted 57.71% of the isolated strains, and *Enterococcus faecalis* belonging to the *Enterococcaceae* family constituted 21.03% of the isolated strains.	Zhu et al. [174]
Placenta/amniotic fluid/meconium	15 mother–infant pairs	16S rRNA gene pyrosequencing, quantitative PCR	● in the amniotic fluid and placenta, the dominant species are *Enterobacter* and *Escherichia/Shigella*, followed by *Cutibacterium* (also detectable in meconium); ● small amount of *Streptococcus* genus in amniotic fluid, placenta, and meconium (<1%); ● relative abundance of the *Streptococcus* genus in colostrum (12%) and infant feces (24%); ● low relative abundance of the *Staphylococcus* genus in amniotic fluid (<1%) and placenta (<1%) compared to meconium (20%); ● *Lactobacillus* genus present in samples of amniotic fluid (1.15%), placenta (<1%), colostrum (2.15%) and meconium (2.53%); ● presence of *Propionibacteria* and *Staphylococci* in the placenta and amniotic fluid; ● at the phylum level, meconium microbiota dominated by *Firmicutes*; ● the *Staphylococcaceae* most frequently detected in meconium samples.	Collado et al. [175]
Placenta	34 women	PCR	● presence of *Bifidobacterium* spp. in 25 placenta samples from vaginal delivery and 8 placenta samples from cesarean section; ● presence of *L. rhammosus* in 23 placenta samples from vaginal delivery and 8 placenta samples from cesarean section; ● in 5 placenta samples from vaginal delivery, a representative colony of *Staphylococcus* spp., *Corynebacterium* spp., and *Lactobacillus crispatus*; ● in 1 placenta sample from a cesarean section, a representative colony of *Clostridium* spp.	Satokari et al. [179]
Meconium	301 newborns	16S rRNA gene sequencing	● dominant: *Firmicutes*, *Proteobacteria*, *Actinobacteria*, *Cyanobacteria* and *Bacteroidetes.*	Dong et al. [180]
Meconium	21 newborns	PCR	● dominant genera *Enterococcus* and *Staphylococcus.*	Jiménez et al. [41]
Meconium	330 very preterm infants (gestational ages 28 to 32 weeks)	16S rRNA gene sequencing	● dominant genera *Bifidobacterium*, *Staphylococcus* and *Enterococcus.*	Klopp et al. [181]
Meconium	117 preterm neonates	16S rRNA gene sequencing, PCR	● the most numerous phylum *Proteobacteria;* ● the most numerous genus, *Bifidobacterium.*	Morais et al. [182]
Meconium	63 preterm infants born < 33 weeks gestational age	16S rRNA sequencing using PGM	● dominant phylum: *Proteobacteria*, *Bacteroidetes*, *Firmicutes* and *Actinobacteria.*	Terrazzan Nutricionist et al. [183]
Cord blood	20 healthy neonates born by cesarean section.	16S rRNA gene sequencing, PCR	● presence of *Enterococcus faecium*, *Cutibacterium acnes*, *Staphylococcus epidermidis* and *Streptococcus sanguinis.*	Jiménez et al. [177]

DNA—deoxyribonucleic acid, rRNA—ribosomal ribonucleic acid; PCR—polymerase chain reaction.

Yatsunenko et al. indicated that the microbiome of the gastrointestinal tract of children at birth is characterized by greater inter-individual variability but significantly less diversity than the microbiome of the gastrointestinal tract of adults [153]. *Firmicutes*, *Proteobacteria*, and *Actinobacteria* are the dominant phyla in the neonatal gastrointestinal microbiome. However, the dominant phylum in the adult gastrointestinal microbiome is *Bacteroidetes* [184]. Also interesting are the studies of Collado et al. [46], who discovered common features of the microbiota when assessing the amniotic, placental, and meconium microbiota in newborns born by cesarean section. The same study shows that the predominant phylum in the amniotic fluid and placenta is *Proteobacteria*, with *Enterobacter*, *Escherichia*, and *Shigella* being the predominant bacterial genus, followed by *Cutibacterium*. Much smaller amounts of these bacteria were detected in the colostrum, meconium, and feces of infants. The exact mechanism by which these microorganisms are transmitted from mother to fetus is currently unknown, but research shows significant similarities between placental and oral microbiomes [171]. Therefore, it can be assumed that the colonization of microorganisms in the fetal gastrointestinal tract is shaped by the microbiome of the mother’s oral cavity, which is the source of bacterial translocation to the placenta [171,178]. The oral microbiota contains over 600 taxa in 13 phyla, including *Chlamydiae*, *Synergistetes*, *Firmicutes*, *Actinobacteria*, *Bacteroidetes*, *Chloroflexi*, *Proteobacteria*, *Euryarchaeota*, *Fusobacteriia*, *Spirochaetes*, and *Tenericutes* [185]. Studies conducted on pregnant women and 6 weeks after delivery indicate that the diversity of the oral microbiota during pregnancy remains relatively stable. However, pathogenic hormonal fluctuations in pregnant women may affect the composition of the oral microbiome [186]. In turn, oral microorganisms may be related to the intrauterine environment, which may interact with many adverse effects of pregnancy, such as premature birth or a deficit in the child’s neurological development [187].

Many commensal bacteria occur in the placenta and uterus. *Proteobacteria* are dominant [171,174], but the most common single species is *E. coli* [187]. A systematic review of 24 studies of the placental microbiome indicated that the most frequently identified genus was *Lactobacillus* [188], which was also the predominant genus in human milk [189]. *Lactobacillus* spp., which has a protective function, was associated with a healthy vaginal and intestinal microbiome [190]. In turn, the uterine microbiome contains not only *Lactobacillus* bacteria, but also *Firmicutes*, *Bacteriodetes*, *Proteobacteria*, and *Actinobacteria* [191]. The presence of microorganisms in the prenatal period was also confirmed by tests conducted after delivery. Jiménez et al. showed that in the meconium of 21 healthy newborns, the dominant population are the genera *Enterococcus* and *Staphylococcus* [41,175], while at the phylum level the meconium microbiota is dominated by *Firmicutes* [175]. Studies assessing the relationship between the meconium microbiome of 301 newborns and neonatal jaundice seem interesting, showing a relationship between a higher abundance of *Bifidobacterium pseudolongum* and a higher alpha diversity and a lower risk of jaundice in infants born by cesarean section. The same study showed that meconium consists mainly of bacteria of the genus *Firmicutes*, *Proteobacteria*, *Actinobacteria*, *Cyanobacteria*, and *Bacteroidetes* [180]. In turn, Morais et al. showed that extremely premature infants born before 28 weeks of gestation had more *Lactobacillus* bacteria, which dominate the mother’s vaginal microbiota, than infants born after 28 weeks of gestation, regardless of the mode of delivery. This confirms the hypothesis that maternal bacteria originating from the vagina and intestines play an important role in shaping the intestinal microbiota of newborns, and that the transfer of bacteria from mother to child is a controlled and time-bound process [182]. *Enterococcus aecium*, *Cutibacterium acnes*, *S. epidermidis*, and *Streptococcus sanguinis* have been identified in the umbilical cord blood of healthy neonates born by cesarean section [177]. The intestine of newborns is initially dominated by the genera *Bifidobacterium*, *Veillonella*, *Streptococcus*, *Citrobacter*, *Escherichia*, *Bacteroides*, and *Clostridium*, which are also abundant in the intestinal microbiota of adults [192], but eventually they are inhabited by the two dominant groups of anaerobic bacteria *Firmicutes* and *Bacteroidetes* [193].

Despite numerous scientific reports on fetal colonization, Kennedy et al.’s recent studies did not show evidence of the existence of a fetal microbiome, which calls into question previous findings. Kennedy et al. concluded that low microbial biomass and contaminants are responsible for erroneous prior fetal microbiome findings [170].

However, it should be remembered that during pregnancy, the mother’s metabolism and microbiota adapt to the most optimal state for the developing fetus [194,195]. According to the literature, the diversity of vaginal bacterial microbiota is decreasing, but the number of *Lactobacillus* bacteria is increasing [196]. The intestinal microbiota of a pregnant woman also changes. The number of proteobacteria and actinomycetes increases, but the number of bacteria that produce butyrate decreases. There is also a reduction in the diversity of bacteria in the gastrointestinal tract [197]. In turn, during natural childbirth, the baby passing through the birth canal is exposed to a variety of microorganisms that come from the birth canal and the mother’s intestines, as well as from the hospital environment. The formation of the composition of the intestinal microbiota of newborns is a dynamic and slow process. *Lactobacillus* and *Enterobacteria*, as well as other facultative anaerobes, colonize the intestines first, and obligate bacteria, such as *Bifidobacteria* and *Bacteroides*, colonize the intestine within a week of birth. Scientific research has shown that the development of a child’s intestinal microbiota is not stable. In the first year after birth, *Bifidobacteria* predominate, but genera such as *Fusobacterium* and *Ruminococcus Enterococcus*, *Staphylococcus*, *Streptococcus* and *Cutibacterium* have also been identified [177]. The fundamental factor shaping the human intestinal microbiota is diet [198]. The World Health Organization recommends introducing complementary foods after the baby is 6 months old. The introduction of solid foods into a child’s diet supplements the intestinal microbiota, which becomes more diverse and similar to the intestinal microbiota of an adult [199,200]. The latest scientific research highlights the important role that microorganisms play in early life in the maturation and development of infants. Creating a good environment for the growth of various types of microorganisms can influence the proper development of the intestinal microbiota of infants and young children [201]. According to studies, important microbiota include *Saccharomyces boulardii*, *LGG* (*Lactobacillus rhamnosus*), *Lactobacillus reuteri*, and *Bifidobacterium lactis (BB-12*). It cannot also be ignored that probiotics have a significant impact on the development of intestinal microbiota in infants and young children, and also have the ability to prevent diseases such as diarrhea, infections [202], allergies [203], and neonatal colic [204].

## 7. Does the Composition of Human Milk Influence the Formation of a Newborn’s Microbiota?

According to scientific reports, colonization of the gastrointestinal tract in children is significantly influenced by the microbiome of human milk. Therefore, it is important to ensure that, especially in the first 100 days of the child’s life, there are no disturbances of the human milk microbiome, which could consequently contribute to the destruction of the microbiological balance of the child’s intestines [75]. The composition of the human milk microbiome affects the baby’s health in two ways: it facilitates digestive processes and also promotes intestinal immune homeostasis [26]. Bacterial diversity plays a key role in maintaining the immune balance in both children and adults [205]. In particular, bacteria derived from human milk provide early antigenic stimuli that have a beneficial effect on the maturation of the intestinal immune system [26]. Research indicates that approximately 25–30% of children’s intestinal microbiota come from human milk [43]. In exclusively breastfed children, a lower diversity but higher number of *Lactobacillus* spp. *B. breve* and *B. bifidum* bacteria were observed compared to formula-fed children. In turn, cessation of breastfeeding results in faster maturation of the intestinal microbiome, as evidenced by the *Firmicutes* phylum [206]. Primary colonization of *Lactobacillus* spp. and *Bifidobacterium* spp. results in a more acidic intestinal environment with high concentrations of short-chain fatty acids, which are reproduced by bacterial fermentation of human milk oligosaccharides. Therefore, it is important to remember that human milk controls the development of the newborn’s intestinal microbiota indirectly (transfer of prebiotics and promoting the growth of specific bacterial species such as *Bifidobacterium* spp.) as well as directly (vertical transfer of pioneer bacterial species) [26].

Determining the correct profile of intestinal bacteria is important in preventing dysbiosis, as well as minimizing its impact on health [207]. In prematurely born children, NEC is a consequence of an enhanced and devastating inflammatory response to intestinal dysbiosis, resulting in tissue damage and impaired intestinal barrier integrity [208]. Microbial dysbiosis may be a risk factor for the development of diseases not only in the first months of life but also later in childhood. In particular, dysbiosis of the respiratory and digestive tracts may cause the development of asthma and obesity [209]. Moreover, metabolites that are recreated by microorganisms moving from the damaged intestinal barrier can affect several organs, causing systemic metabolic inflammation [63]. Therefore, both diseases of the neonatal period, such as NEC, and diseases that begin later in life, such as asthma and obesity, are clinical symptoms of early microbiota dysbiosis [210].

The impact of breastfeeding on shaping the intestinal microbiota of children is the best researched and documented. However, it should be remembered that breastfeeding also has a significant impact on shaping the microbiota of the oral cavity, nasopharynx, and respiratory tract. This is confirmed by research by Holgerson et al., who showed differences in the oral microbiota between 3-month-old breastfed infants and 3-month-old infants fed formula milk. The presence of *Lactobacillus* species was found in 27.8% of exclusively and partially breastfed infants, but not in formula-fed infants [211]. Subsequent studies indicate that the *Firmicutes* phylum dominated the oral bacterial environment in both groups (breastfed vs. formula-fed infants). A higher prevalence of *Prevotella* and the *Bacteroidetes* phylum was observed in the oral cavity of formula-fed infants compared to breastfed infants. However, a higher frequency of *Proteobacteria* and *Actinobacteria* species was found in the oral microbiota of breastfed children [40]. Analysis of the oral microbiota of infants and colostrum collected from pregnant women before delivery revealed common types of bacteria, with Streptococcus spp. and Staphylococcus spp. being the most abundant. The presence of typical oral microbiota in colostrum analyzed before birth, i.e., before the contact of the mammary gland with the newborn, suggests that breastfeeding is one of the first microbiological sources for the formation of the child’s oral microbiota [39]. In turn, a study aimed at comparing the microbiome of human milk and the microbiome of oral and fecal samples of healthy breastfeeding women and their infants showed that variability of the human milk microbiome may have an impact on the microbiome of the infants’ gastrointestinal tract. Additionally, the most abundant genera in both mother and infant oral samples were the genera *Streptococcus*, *Staphylococcus*, *Gemella*, *Rothia*, and *Veillonella* [212]. Dzidic et al. showed that shorter breastfeeding habits during the first two years of a child’s life were associated with a distinct oral bacterial composition later. The results of these studies also describe the development of oral microbiota as ecological succession. Altered bacterial colonization patterns during the first year of life may have long-lasting effects on both the oral and systemic health of children. Changing the pattern of bacterial colonization during the first year of life can have long-lasting effects on both the oral and systemic health of the child [213].

Scientific research shows that breastfeeding reduces the risk of respiratory infections in the neonatal and infancy period. It has been proven that breastfed children get sick less frequently than formula-fed children, and that the duration of infection is shorter and its severity is weaker. Biesbroek et al., analyzing the nasopharynx microbiota in 6-week-old infants, observed a significant difference in the microbiological composition between children fed with formula and breastfed children [214]. Formula-fed children had lower numbers of lactic acid bacteria and increased numbers of *Staphylococcus* spp. and anaerobic bacteria, such as *Prevotella* and *Veillonella* species. In turn, the respiratory microbiota of breastfed infants showed low abundance of the following genera: *Veillonella*, *Prevotella*, *Streptococcus*, *Rothia*, *Gemella*, and *Granulicatella* compared to children who were fed with formula milk. Interestingly, the occurrence of the *Dolosigranulum* genus was reported to be inversely related to the symptoms of wheezing and the number of previous respiratory infections. However, based on the research of Biesbroek et al., it can be concluded that at the age of 6 months, the connection between breastfeeding and the composition of the nasopharyngeal microbiota disappears [214,215].

## 8. Microbiome Assessment Methods

**Figure 4:**
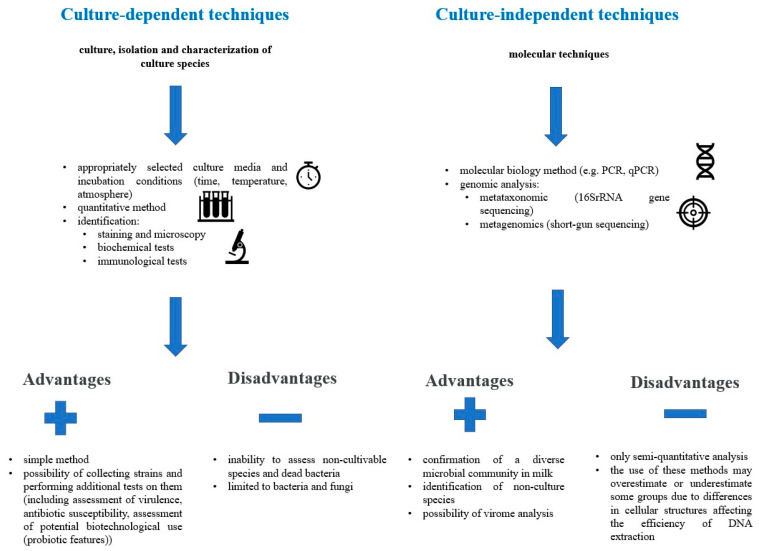
Methods for assessing the human milk microbiome, including advantages and disadvantages.

So far, research on the assessment of the presence of microorganisms in human milk has been conducted using classic microbiological methods, including cultures and identification using biochemical and immunological methods. The development of molecular biology techniques allowed the use of new methods, including mainly the PCR reaction with its variants and sequencing (Figure 4). The use of new techniques has allowed for the confirmation of a rich and diverse microbial community (including non-cultured ones) in human milk samples [50,78,216]. So far, it has been shown that culture methods can estimate the number of bacteria at the level of 10^2^ to 10^4^ CFU/mL, while the use of culture-independent methods may reveal a bacterial titer at the level of 10^4^ to 10^5^ CFU/mL, which may indicate the presence of dead bacteria and non-culturable species [49]. The first study of the milk microbiome using pyrosequencing showed that lactic acid bacterial communities are generally complex [50].

Using breeding methods, Jost et al. showed that the average number of live bacteria in human milk was low and was at the level of 3 log CFU/mL [78]. This is consistent with the results presented, among others, by Perez et al. and Heikkila et al. [51]. In turn, Solis et al. showed that the average bacterial values in the milk of healthy women were 3–5 log CFU/mL [68]. Discrepancies between studies may result from the microbiological media used, culture conditions, and other factors influencing the formation of the milk microbiome, including geographic location.

Both breeding and molecular biology methods have certain limitations. Obtaining different results between researchers may result from different protocols for sample collection and preservation (e.g., aseptic methods, time of day, breast cleaning methods, collecting samples before or after feeding, manual expression or expression with a breast pump) and, at a later stage, DNA extraction (e.g., from whole or skimmed milk, use of commercial kits, enzymatic lysis), selection of specific primers, and sequencing platforms [50,78,216]. It is desirable that standardized protocols for sample collection and validation of the methods used be developed in the future. Due to the composition of cell walls, DNA isolation methods, and the number of reference gene copies, the total bacterial count may be overestimated or underestimated [33].

Sequencing is unable to distinguish live from dead microbes or from cell-free DNA, so viability dyes, such as propidium monoazide, are used [217]. Another limitation is that it is impossible to obtain quantitative results using this method, and, at best, only semi-quantitative data can be obtained.

As recommended by Jimenez et al., testing of human milk samples should be preceded by blind control sequencing. If such control is not carried out, the results obtained should be analyzed with caution [33]. In turn, Soto et al., comparing the results from breeding techniques and sequencing, showed high consistency of the results obtained, both in relation to the genus and species [71].

## 9. Perspectives

Information about microorganisms in human milk has been limited so far. There has been an increase in research on this topic in recent years. However, the origin of these populations in milk is not fully understood and remains the subject of much debate. Moreover, the biological role of neonatal health in the short and long term has not yet been determined. The fundamental task for scientists working with human milk is to define a healthy microbiota and prevent microbial dysbiosis, which is crucial in the first months of a child’s life and also influences the development of chronic diseases, such as obesity and asthma. The methods of collecting milk samples and assessing microorganisms in milk should also be discussed. The topic of the milk microbiome is still relevant and requires research in various aspects by scientists around the world.

## Figures and Tables

**Table 1:** Diversity of the human milk microbiome depending on geographical location.

Geographical Localization	Number of Women; Residence	Microbiota Analysis Method	Microbiota Diversity	References
Africa (Mozambique)	121 samples of milk; Manhiça town and the surrounding villages	qPCR; culture	● the most frequent genera isolated by culture were *Staphylococcus* (96.4%), *Streptococcus* (92.7%), and *Lactobacillus* (56.4%); ● the most frequent groups identified by PCR were *Streptococcus* (94.8%), *Staphylococcus epidermidis* (85.4%), *Enterococcus* spp. (83.9%), and *Bifidobacterium* spp. (81.8%); ● women who exclusively breastfed had a higher percentage of *Streptococcus parasanguis* compared to women who also used formula; ● women who were HIV positive had a higher absolute number of *Lactobacillus* spp.	González et al. [98]
Africa (Republic of Burundi)	30 women; urban	16S rRNA gene sequencing	● predominance of *Aquabacterium*, *Serratia* and *Peptrostreptococcus* in colostrum and *Rhizobium*, *Dolosigranulum* and in mature milk.	Drago et al. [104]
Africa (South Africa)	20 women; urban	PCR and sequencing	● higher abundance of *Proteobacteria* in South Africa women compared to women from Finland, Spain, and China; ● *Bifidobacteriaceae* was found in women from South Africa, while no *Bifidobacteriaceae* was found in women from Finland, Spain and China.	Kumar et al. [102]
Asia (China)	20 women; urban	PCR and sequencing	● higher abundance of *Streptococcus* spp. in Chinese women compared to women from Finland, Spain and South Africa; ● *Enterococcaceae* was found in samples from women from South Africa, Finland, and Spain, while no *Enterococcaceae* was found in women from China.	Kumar et al. [102]
Asia (China)	90 women (60 samples without special meaning and 30 samples collected aseptically); urban	16S rRNA gene sequencing; qPCR for total bacteria loads	● dominant species: *Staphylococcus* and *Streptococcus*; ● higher number of bacteria in milk collected using a standard protocol than in samples collected aseptically; ● low abundance of *Bifidobacterium* spp. and LAB; ● high number of *Acinetobacter* spp. (~32%) in samples collected using a standard protocol.	Sakwinska et al. [105]
Asia (China, 11 cites)	89 women; urban	16S rRNA gene sequencing; qPCR	● the dominant composition of the microbiome (in all regions) included the following species: *Staphylococcus* (100% of samples), *Bacillus* (87%), *Enterococcus* (76%), *Streptococcus* (76%), *Lactobacillus* (40%); ● the composition of the microbiome was region-specific: samples from women in the northwest and north of China showed greater diversity compared to other regions.	Ding et al. [106]
Asia (India)	50 women (32 with mastitis); urban	16S rRNA gene sequencing	● the dominant phyla are: *Proteobacteria* (50%) and *Firmicutes* (17%); ● at the genus level, control subjects had relatively more *Acinetobacter*, *Ruminococcus*, *Clostridium*, and *Eubacterium* compared to other groups; ● higher numbers of the following genera were found in the milk of women with mastitis: *Aeromonas*, *Staphylococcus*, *Klebsiella*, *Ralstonia*, *Bacillus*, *Pantoea*, *Serratia*, *Enterococcus*, and *Pseudomonas*.	Patel et al. [107]
Asia (Syria)	15 samples; villages	MALDI-TOF MS; 16S rRNA gene sequencing	● 36 different species of the genus: *Lactobacillus*, *Enterococcus*, *Weissella*, *Streptococcus*, *Staphylococcus*, and *Pediococcus.*	Albesharat et al. [108]
Asia (Taiwan)	30 women; urban	PCR; culture	● the number of bacteria in milk ranged from 4.0 × 10^1^ to 7.1 × 10^5^ CFU/mL; ● the presence of antibiotic-resistant strains of the following species: *Staphylococcus*, *Streptococcus*, *Enterococcus*, and *Acinetobacter* in milk samples.	Huang et al. [109]
Asia (Taiwan)	19 women; urban	Culture	● approximately 20 types of bacteria were isolated, including *Staphylococcus* (6 species), *Streptococcus* (4 species), *Enterococcus* (2 species), *Lactobacillus* (1 species), and bacteria belonging to other genera (7 species); ● potentially pathogenic species present in milk: *Kluyvera ascorbata*, *Klebsiella oxytoca*, *Klebsiella pneumoniae*, *Acinetobacter baumannii*, *Actinomyces bovis*, and *Staphylococcus aureus*; ● the presence of bacteria resistant to antibiotics, including last resort, was demonstrated.	Chen et al. [110]
Europe (Finland)	40 women; urban	Culture; identification with RFLP	● milk microbiome dominated by *Staphylococcus* spp. (64%), *Streptococcus* spp. (30%); ● LAB in 13% of the samples.	Heikkilä et al. [111]
Europe (Finland, Spain)	20 women; urban	PCR and sequencing	● higher abundance of *Bacteroidetes* in Spanish women (natural childbirth) compared to women from South America and China; ● higher abundance of *Firmicutes* among women from Finland compared to women from South Africa and China; ● higher abundance of *Cutibacterium* and *Pseudomonas* spp. in Spanish women compared to women from South Africa and China.	Kumar et al. [102]
Europe (Italy)	20 women; urban	16S rRNA gene sequencing	● colostrum and mature milk showed high bacterial counts: > 200 generations/sample; ● higher relative abundance of anaerobic bacteria in mature milk compared to colostrum; ● predominance of *Abiotrophia* and *Alloiococcus* in colostrum and *Parabacteroides* in mature milk.	Drago et al. [104]
Europe (Italy)	36 women; urban	16S rRNA gene sequencing; IluminaMiSeq	● diverse profile of breast milk microbiota; ● dominant genus: *Streptococcus*, *Staphylococcus*, *Bifidobacterium*; ● the microbiota of breast milk was more diverse than the oral cavity and feces of breastfed infants.	Biagi et al. [112]
Europe (Slovenia)	45 women; urban	PCR; DGGE; qPCR	● the dominant genera in colostrum are *Staphylococcus* and *Streptococcus;* ● high abundance of *Enterobacteriaceae* (100%), *Clostridia* (95.6%), *Bacteroides-Prevotella* (62.2%), and *Bifidobacterium* (53.3%) was confirmed in colostrum samples; ● *Enterococcus* spp. detected in 8.9% of colostrum samples.	Obermajer et al. [113]
Europe (Spain)	20 women (10 healthy, 10 mastitis); urban	MALDI-TOF MS; Shotgun sequencing	● among the bacterial sequences, the dominant phyla were *Proteobacteria*, *Firmicutes* and *Bacteroidetes*; ● a healthy core microbiome included the genera *Staphylococcus*, *Streptococcus*, *Bacteroides*, *Faecalibacterium*, *Ruminococcus*, *Lactobacillus*, and *Cutibacterium*; ● *S. aureus* predominated in samples from women with acute mastitis, and *S. epidermidis* predominated in the milk of women with subacute mastitis.	Jiménez et al. [33]
Europe (Spain, Madrid)	10 women (5 mothers were born by vaginal delivery and 5 mothers by cesarean section); urban	16S rRNA gene sequencing	● the most frequently isolated species of the *Staphylococcus* genus was *S. epidermidis*, and of the *Streptococcus* genus—*S. mitis*; ● the dominant LAB species were *Leuconostoc citreum* and *Lactococcus lactis*.	Martín et al. [47]
Europe (Spain, Gijon)	20 women; urban	16S rRNA gene sequencing	● the most frequently isolated species of the *Staphylococcus* genus was *S. epidermidis*, and of the *Streptococcus genus—S. salivarius*; ● 5% of all isolates belonged to the genus *Lactobacillus*, and another 5% were *Bifidobacterium* spp.; ● the most frequently isolated LAB was *Lactobacillus gasseri*.	Solís et al. [68]
Europe (Switzerland)	7 women; urban	16S rRNA gene sequencing; anaerobic culture	● dominant species: *Staphylococcus*, *Streptococcus*, *Cutibacterium*; ● the presence of obligate anaerobes of the genera *Bifidobacterium*, *Veillonella*, and *Bacteroides*, as well as those synthesizing butyrate, i.e., *Faecalibacterium* spp. and *Roseburia* spp.	Jost et al. [78]
North America (Canada)	10 women; urban	Illumina sequencing	● dominant phyla: *Proteobacteria* (65%) and *Firmicutes* (34%); ● at the genus level: 75% *Staphylococcus*, 15% *Pseudomonas*, 2% *Edwardsiella*, and 1% *Pantoea*, *Treponema*, *Streptococcus*, and *Campylobacter*, respectively.	Ward et al. [32]
North America (Canada)	39 Caucasian Canadian women recruited from London, Ontario, and the surrounding area	MALDI-TOF MS	● higher number of bacteria in colostrum than in mature milk; ● *L. gasseri* only detected in women of normal weight and who delivered vaginally.	Urbaniak et al. [99]
North America (Haiti)	50 women (25 HIV-positive mothers and 25 HIV-negative mothers); urban	16S rRNA gene sequencing	● no differences in the microbiome between HIV-positive and HIV-negative women.	Bender et al. [114]
North America (USA)	16 women; urban	16S rRNA gene sequencing	● the most numerous genera in milk were *Streptococcus*, *Staphylococcus*, *Serratia*, and *Corynebacterium*; ● eight other genera accounted for ≥1% of the communities observed in the samples.	Hunt et al. [50]
North America (USA)	12 women; urban	16S rRNA gene sequencing; IluminaHiSeq; culture	● Genera: *Acinetobacter*, *Staphylococcus*, *Halomonas*, *Bacillus*, *Stenotrophomonas*, unclassified genus *Enterobacteriaceae*, *Streptococcus*, *Shewanella*, *Pseudomonas*, *Serratia*, *Enterococcus*, unclassified genus *Methylobacteriaceae*, unclassified genus *Pseudomonadaceae*, unclassified genus *Xanthomonadaceae*, and *Bacteroides* accounted for 85% of the sequences found in the milk of donor mothers; ● the most abundant genera in breast milk were *Halomonas*, *Staphylococcus*, *Shewanella*, *Corynebacterium*, genus *Enterobacteriaceae*, *Acinetobacter*, unclassified genus *Methylobacteriaceae*, unclassified genus *Enterobacteriaceae*, *Bacteroides*, *Stenotrophomonas*, and *Lactobacillus*.	Cacho et al. [115]
South America (Brazil)	47 women (of which two with mastitis); urban	MALDI-TOF MS	● the total number of bacteria in breast milk ranged from 1.5 to 4.0 log_10_ CFU/mL; ● high number of bacteria in colostrum; ● among LAB the following were isolated: *L. gasseri*, *Bifidobacterium breve*, and *S. salivarius.*	Damaceno et al. [116]
South America (Mexico)	10 women; urban (low-income families)	16S rRNA gene sequencing	● the dominant genera in breast milk are: *Streptococcus*, *Staphylococcus* and *Neisseria*.	Davé et al. [100]

PCR—polymerase chain reaction; MALDI-TOF MS—matrix-assisted laser desorption/ionization time of flight mass spectrometry; LAB—lactic acid bacteria; USA—the United States of America; DGGE—denaturing gradient gel electrophoresis; RFLP—restriction fragment length polymorphism; CFU—colony-forming unit; HIV—human immunodeficiency virus.
